# Supplementary material for: Single-cell microRNA sequencing method comparison and application to cell lines and circulating lung tumor cells
Source: Nat Commun. 2021 Jul 14;12:4316. doi: 10.1038/s41467-021-24611-w (PMC8280203; doi:10.1038/s41467-021-24611-w)
Supplement: Supplementary file 1 — Supplementary Information [file 41467_2021_24611_MOESM1_ESM.pdf]

SUPPLEMENTARY INFORMATION

Single-cell microRNA sequencing method comparison and application to cell lines and circulating lung tumor cells

Sarah M. Hücker, Tobias Fehlmann, Christian Werno, Kathrin Weidele, Florian Lüke, Anke Schlenska-Lange, Christoph A. Klein, Andreas Keller & Stefan Kirsch

Supplementary Figures 1-13

Supplementary Tables 1-5

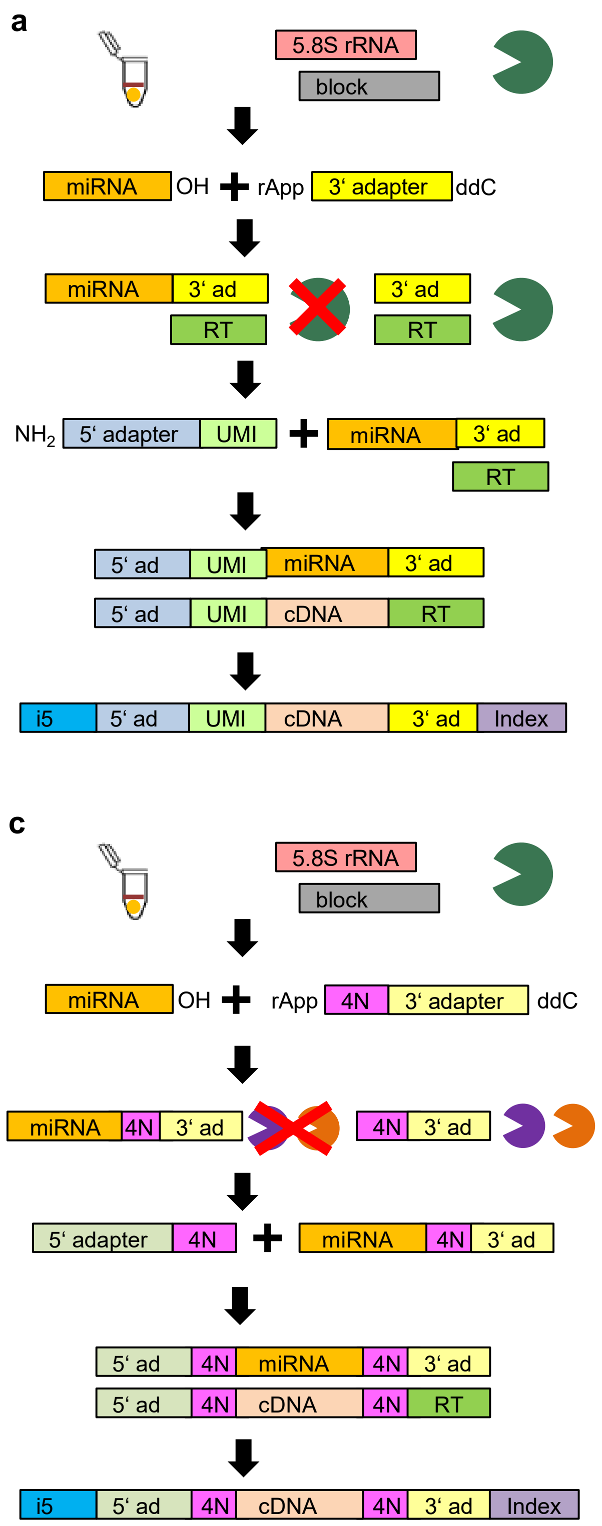

Supplementary Figure 1: Principle of the four miRNA-Seq protocols used. **a,** Overview of the SB and SBN protocol. First, single cells are isolated by micromanipulation and deposited into lysis buffer. A blocking oligo against 5.8S rRNA

was added and the double stranded product was digested with exonuclease. Then, the preadenylated 3’ adapter is ligated. In order to remove unligated 3’ adapter, the reverse transcription primer is added and the double stranded product is digested by exonuclease. Next, the 5’ adapter is ligated, reverse transcription is performed, and the cDNA is amplified in two subsequent PCR reactions. **b,** Overview of the CL protocol. After cell lysis and 5.8S rRNA digest (analogous to SB/SBN protocol), the 3’ adapter and the 5’ adapter are ligated subsequently. The 3’ adapter has a 5’ methyl-phosphonate and the 5’ adapter a 3’ 2-O-methylation modification. These chemical modifications should inhibit adapter dimer formation. Then, the miRNA is reverse transcribed into cDNA and the Illumina adapters are added in two subsequent PCR amplifications. **c,** Overview of the 4N protocol. After cell lysis and 5.8S rRNA digest, the 3’ adapter is ligated. To get rid of unligated 3’ adapter, single strand binding protein, 5’ deadenylase, and an exonuclease are added subsequently. Next, the 5’ adapter is ligated, reverse transcription is performed, and the cDNA is amplified. **d,** Overview of the CATS protocol. After lysis, the miRNA is polyadenylated. Reverse transcription is performed using an oligo-dT primer and a reverse transcriptase, which adds non-templated cytosine nucleotides to the synthesized cDNA. A template switching oligo is used for second strand synthesis. In a PCR amplification the Illumina primers are introduced.

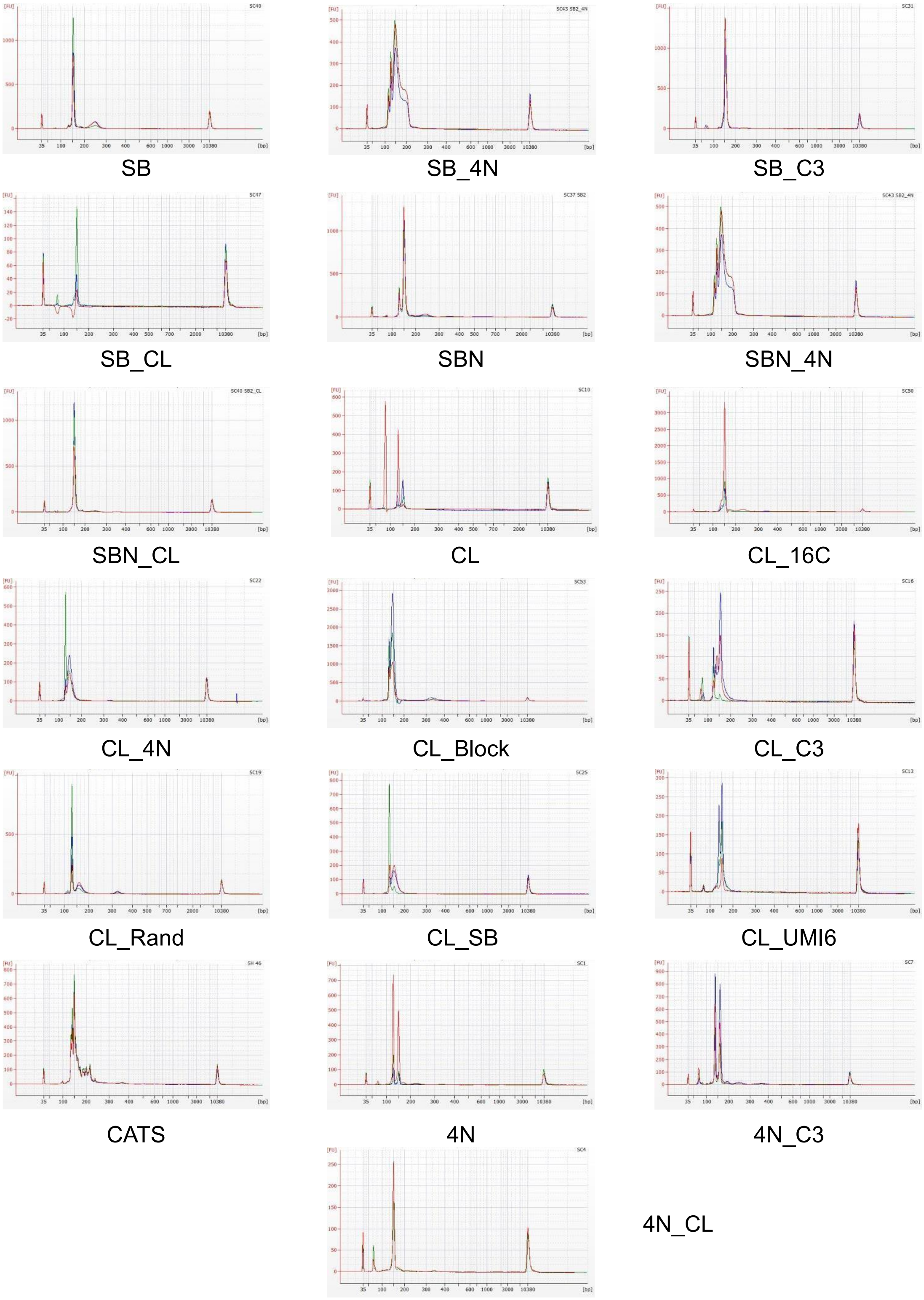

Supplementary Figure 2: Fragment length distribution of the spike-in experiment libraries. If necessary, the libraries were diluted to 1.8 ng/µl and 1 µl was measured on a Bioanalyzer High Sensitivity Chip. The Bioanalyzer profiles of the first three replicates

are overlayed. Fragments of around 125 bp are likely to represent adapter dimers, whereas fragments of 140-150 bp are likely to represent libraries with miRNA insert.

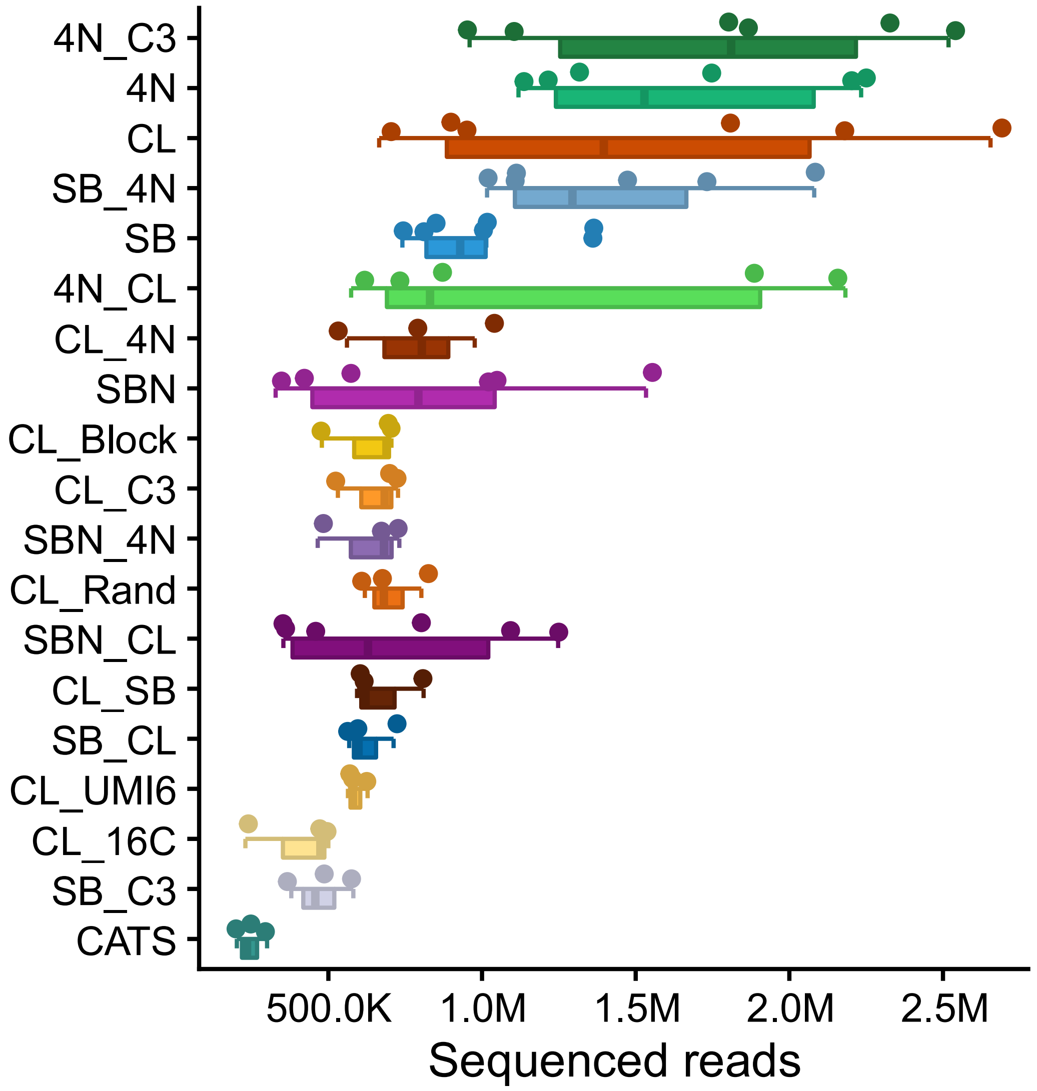

Supplementary Figure 3: Number of reads sequenced in stage 1 grouped by protocol and ordered by decreasing average, shown as boxplot (bottom) and dot plot (top). Each dot represents one sample and is colored according to its protocol. The boxes span the first to the third quartile with the vertical line inside the box representing the median value. The whiskers show the minimum and maximum values or values up to 1.5 times the interquartile range below or above the first or third quartile if outliers are present.

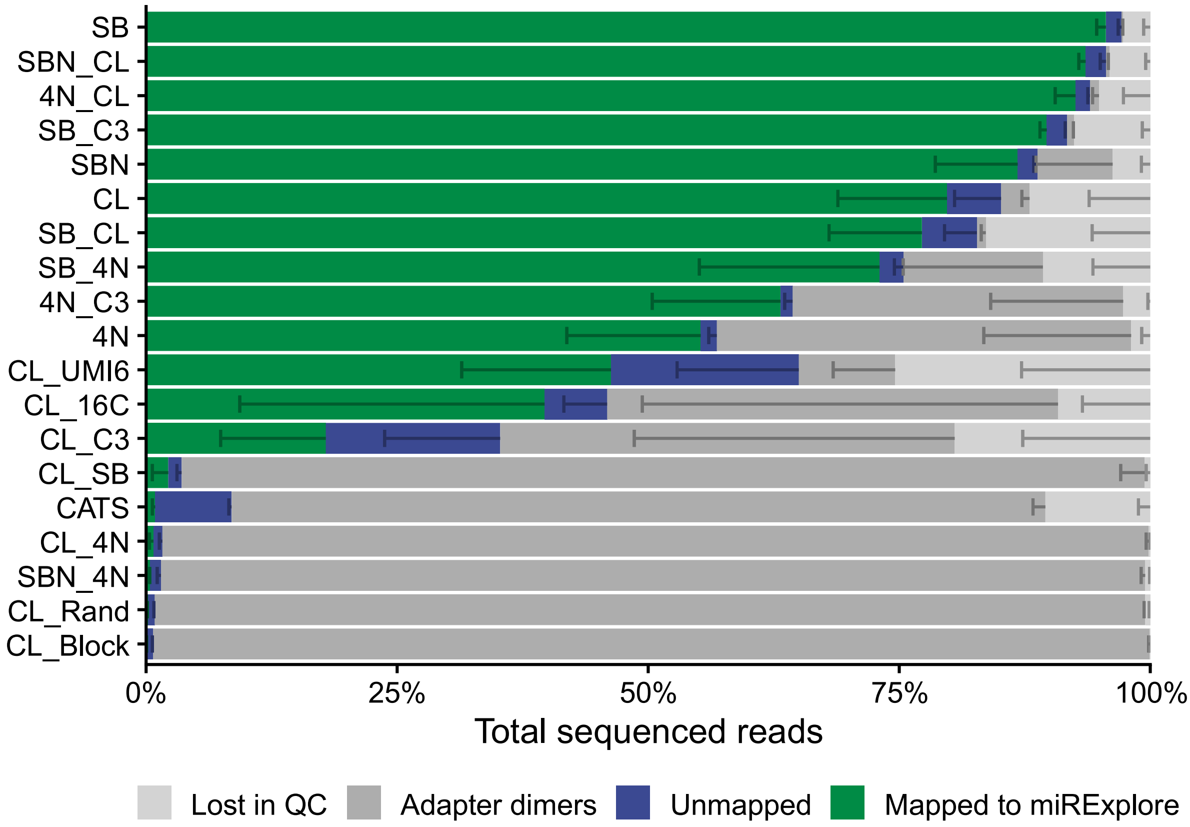

Supplementary Figure 4: Read distribution sorted by decreasing proportion of reads mapping to miRXplore. The data are presented as mean values +/- the standard deviation (n=6 biologically independent samples for the top 8 protocols, n=3 for the others), which is shown as smaller error bar in a darker color than its corresponding read group and only represented in one direction.

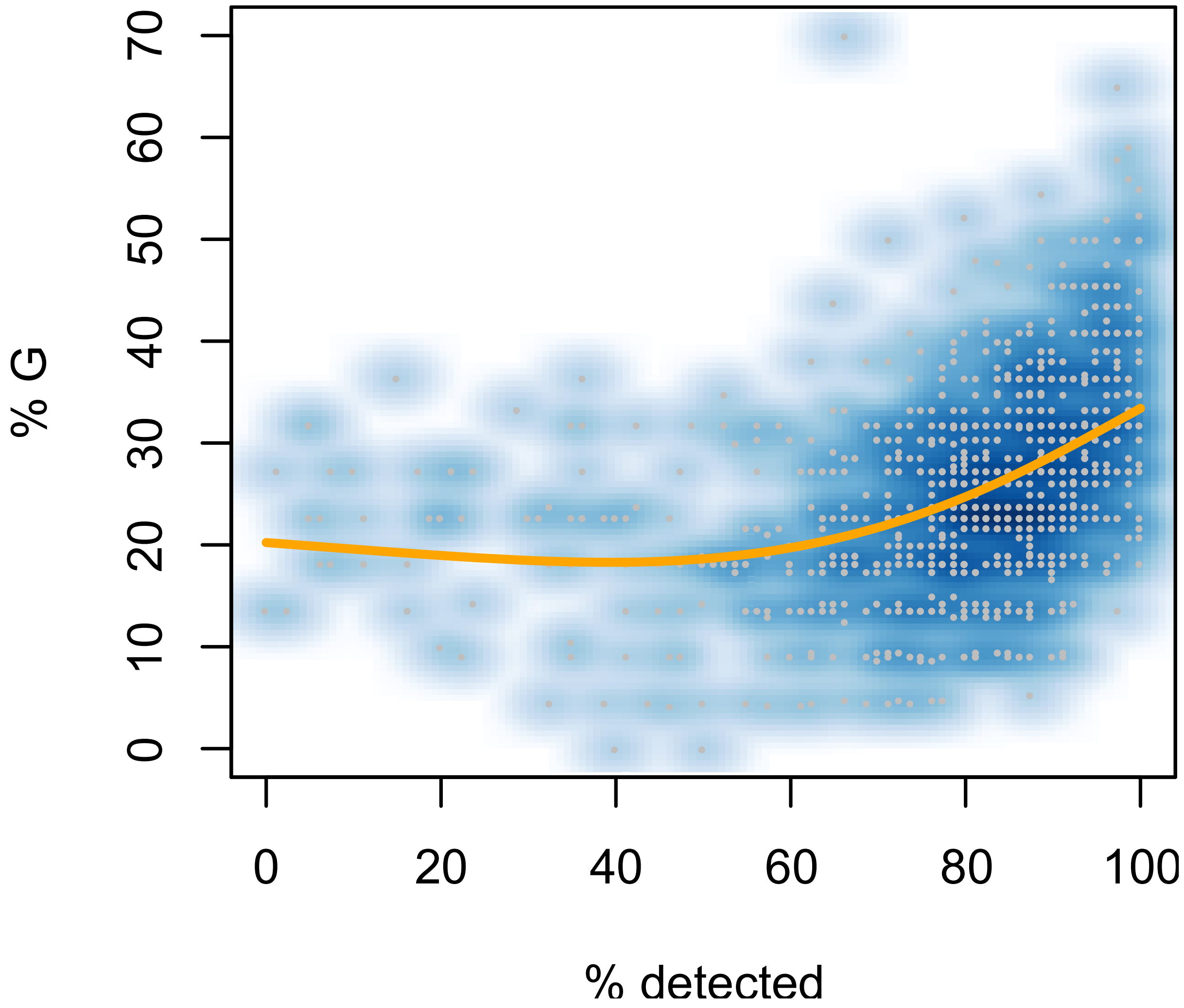

Supplementary Figure 5: Influence of miRNA sequence G-content on detection rate. Each sequence is presented by a grey dot and the density of points is indicated by the blue color gradient. The orange line represents a smoothed spline.

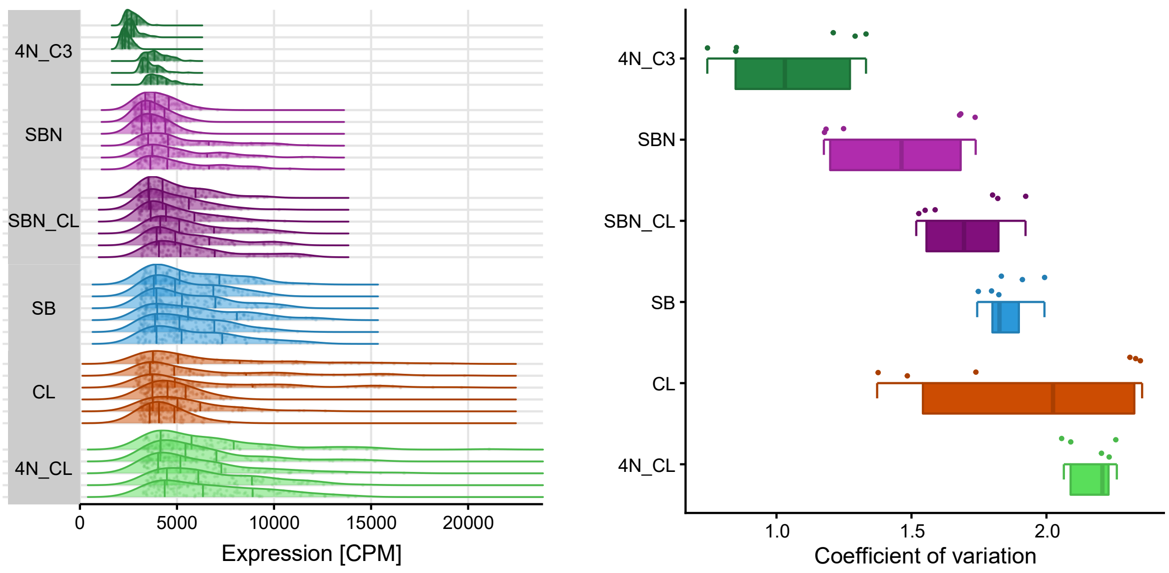

Supplementary Figure 6: Accuracy evaluation of the top 6 UMI protocols based on the derived molecule counts. **a,** Distribution of the top 100 most expressed miRXplore sequences per sample. The samples are grouped by protocol and ordered by ascending coefficient of variation. The vertical lines inside the areas delimit the quartiles. Every dot inside the area represents the expression level of one sequence. The expression level is shown as counts per million (CPM). **b,** Coefficient of variation for all samples grouped by protocol in ascending order shown as dot plot (top) as well as boxplot (bottom). Each sample is represented by a dot. The boxes span the first to the third quartile with the vertical line inside the box representing the median value. The whiskers show the minimum and maximum values or values up to 1.5 times the interquartile range below or above the first or third quartile if outliers are present.

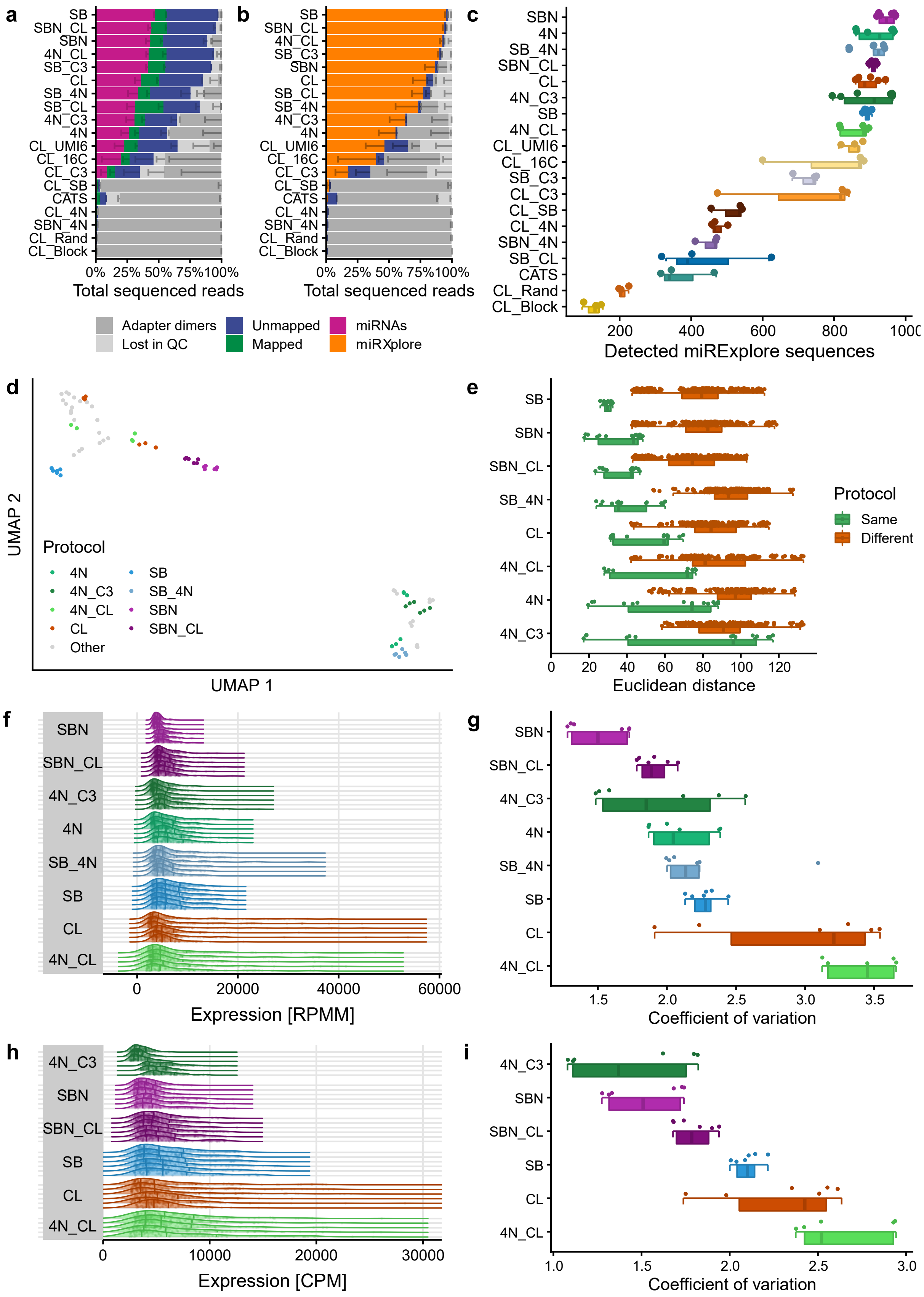

Supplementary Figure 7: Protocol comparison with miRXplore spike-in on all samples subsampled to 300,000 reads. All boxplots are defined as follows: The boxes

span the first to third quartile with the vertical line inside the box representing the median value. The whiskers show the minimum and maximum values or values up to 1.5 times the interquartile range below or above the first or third quartile if outliers are present. Read distribution for all tested protocols, sorted by **a**, miRNA reads proportion and **b**, reads mapping to the miRXplore sequences. The data are presented as mean values +/- standard deviation (n=6 biologically independent samples for the top 8 protocols, n=3 for the others), which is shown as smaller error bar in a darker color and only represented in one direction. **c,** Detected miRXplore sequences for every sample, sorted by decreasing average per protocol, shown as boxplot (bottom) and dot plot (top). Each sample is shown as one dot and colored by protocol. **d,** UMAP embedding of all samples with miRXplore spike-in. The samples of the best 8 protocols are colored, while the remaining protocols are greyed out. **e**, Euclidean distance on the $\log_2$ transformed expression showing the reproducibility between all replicates of the same protocol (green) and between all samples of one protocol variant compared to all other protocol variants (brown). Each dot represents the distance observed between two samples. For each protocol a dot plot (top) and boxplot (bottom) are shown. **f,** Distribution of the top 100 highest expressed miRXplore sequences per sample, normalized as reads per million mapped (RPMM). The samples are grouped by protocol and ordered by ascending coefficient of variation. The vertical lines inside the areas delimit the quartiles. Every dot inside the area represents the expression level of one sequence. **g,** Coefficient of variation for all samples grouped by protocol in ascending order, shown as dot plot (top) and boxplot (bottom). Each sample is represented by a dot. **h,** Same as **f**, showing the distribution of the expressed miRXplore sequences after deduplication, for the top 6 UMI protocols, normalized as counts per million (CPM). **i,** Same as **g**, showing the coefficient of variation after deduplication, for the top 6 UMI protocols.

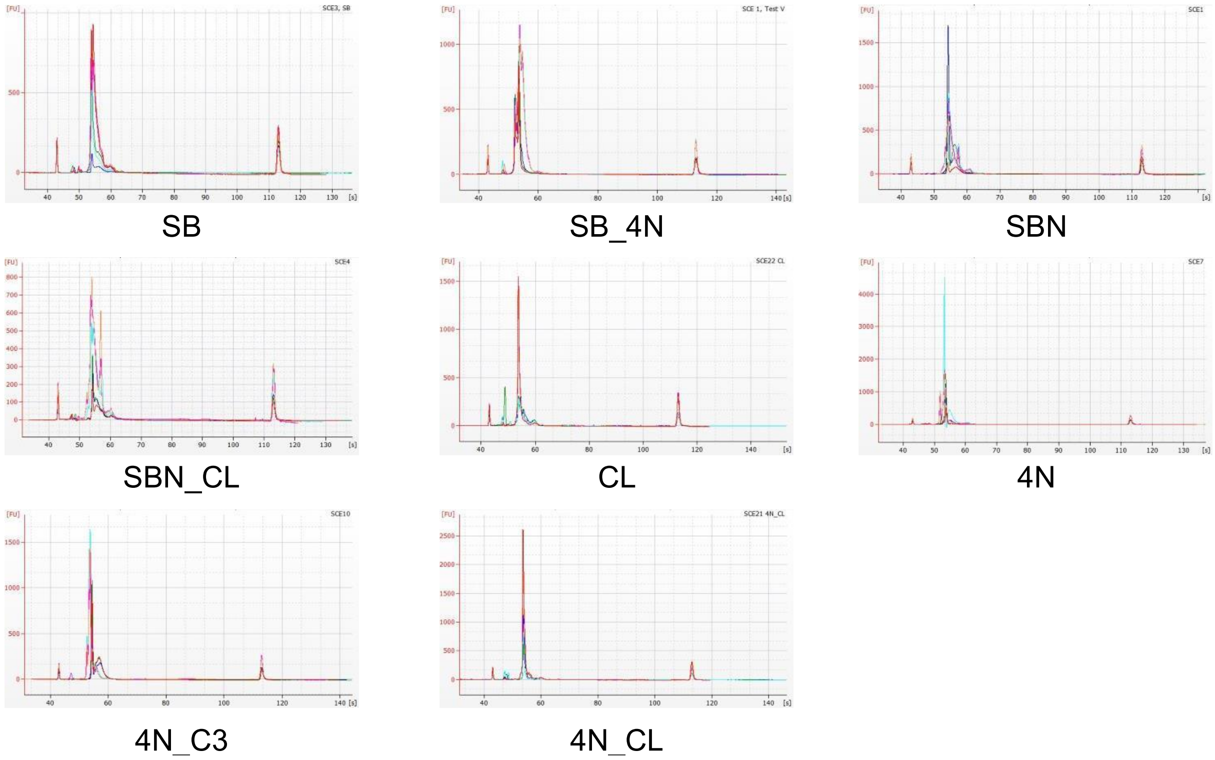

Supplementary Figure 8: Fragment length distribution of the MCF7 single cell equivalent experiment libraries. If necessary, the libraries were diluted to 1.8 ng/µl and 1 µl was measured on a Bioanalyzer High Sensitivity Chip. The Bioanalyzer profiles of all six replicates are overlayed.

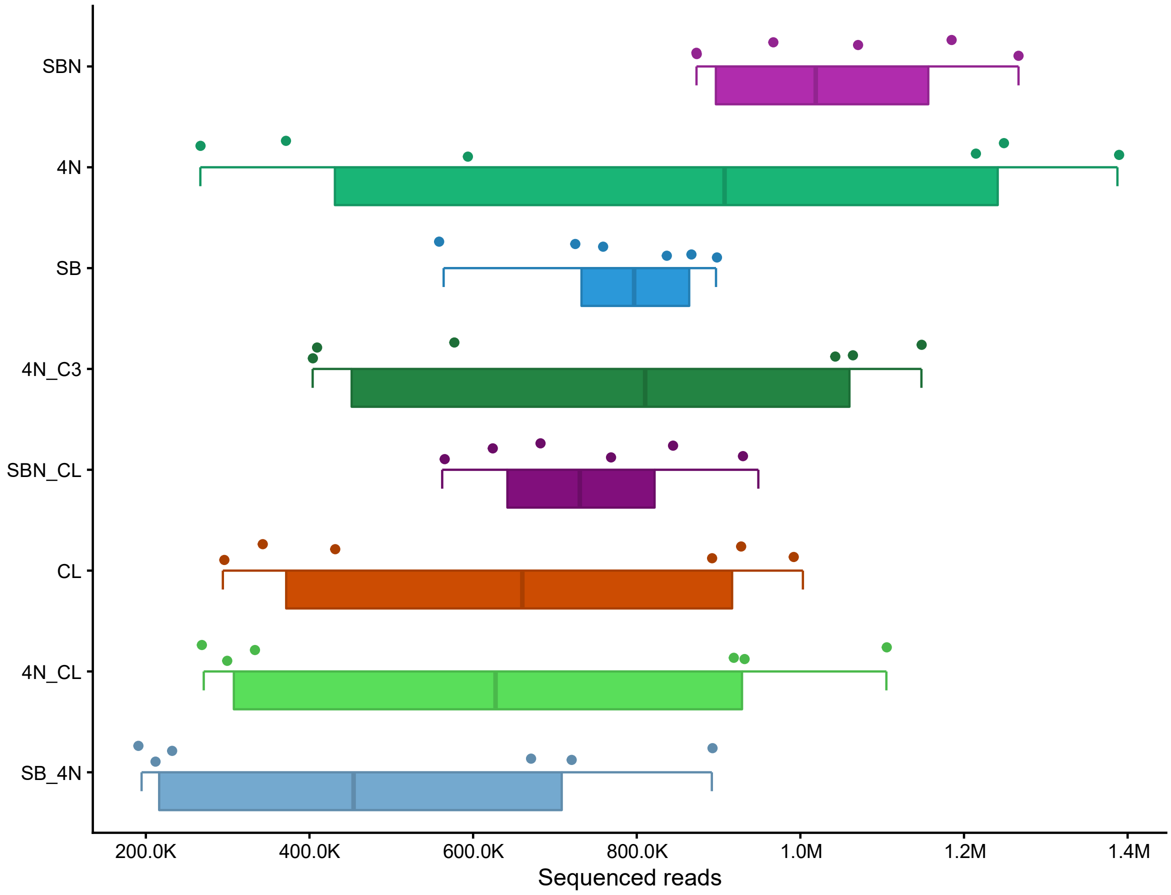

Supplementary Figure 9: Number of reads sequenced in stage 2 grouped by protocol and ordered by decreasing average, shown as boxplot (bottom) and dot plot (top). Each dot represents one sample and is colored according to its protocol. The boxes span the first to the third quartile with the vertical line inside the box representing the median value. The whiskers show the minimum and maximum values or values up to 1.5 times the interquartile range below or above the first or third quartile if outliers are present.

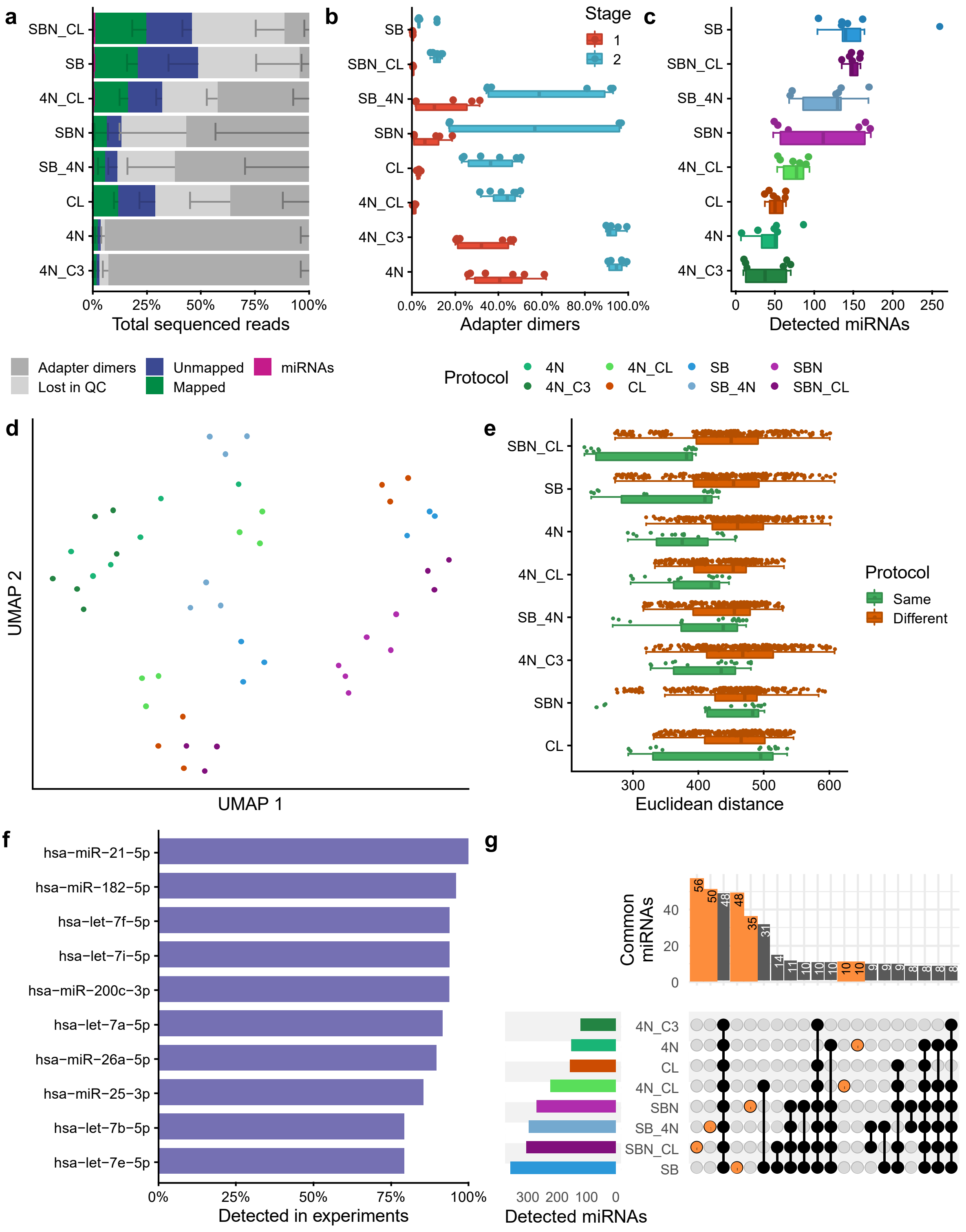

Supplementary Figure 10: Protocol comparison on single cell equivalents on all samples subsampled to 300,000 reads. **a,** Read distribution for all tested protocols, sorted by miRNA reads proportion. The data are presented as mean values +/- the standard deviation (n=6 biologically independent samples), which is shown as smaller

error bar in a darker color than its corresponding read group and only represented in one direction. **b,** Adapter dimers found in stage 1 and stage 2, shown as boxplot (bottom) and dot plot (top). The boxes span the first to third quartile with the vertical line inside the box representing the median value. The whiskers show the minimum and maximum values or values up to 1.5 times the interquartile range below or above the first or third quartile if outliers are present. **c,** Detected miRNAs for every sample, sorted by decreasing average per protocol shown as boxplot (bottom) and dot plot (top). Every dot is a sample colored by protocol. The boxplot was defined in the same manner as for panel **b. d,** UMAP embedding of all samples. Each sample (dot) is colored by its protocol. **e,** Euclidean distance on the $\log_2$ transformed sequence expression showing the reproducibility between all replicates of the same protocol (green) and between all samples of different protocols (brown). Each dot represents the distance observed between two samples. For each protocol a dot plot (top) and a boxplot (bottom) are shown. The boxplot was defined in the same manner as for panel **a**. **f,** Top 10 miRNAs detected in multiple experiments. **g,** Upset plot showing the miRNAs jointly detected in at least one replicate of multiple protocols (black), or exclusively found in only one protocol (orange). The bar plot at the top shows on the y-axis the number of miRNAs detected by the protocols highlighted by connected black or orange dots in the grid below. The bar plot on the left shows on the x-axis the total number of miRNAs detected in a least one of the replicates of the protocol shown on the y-axis.

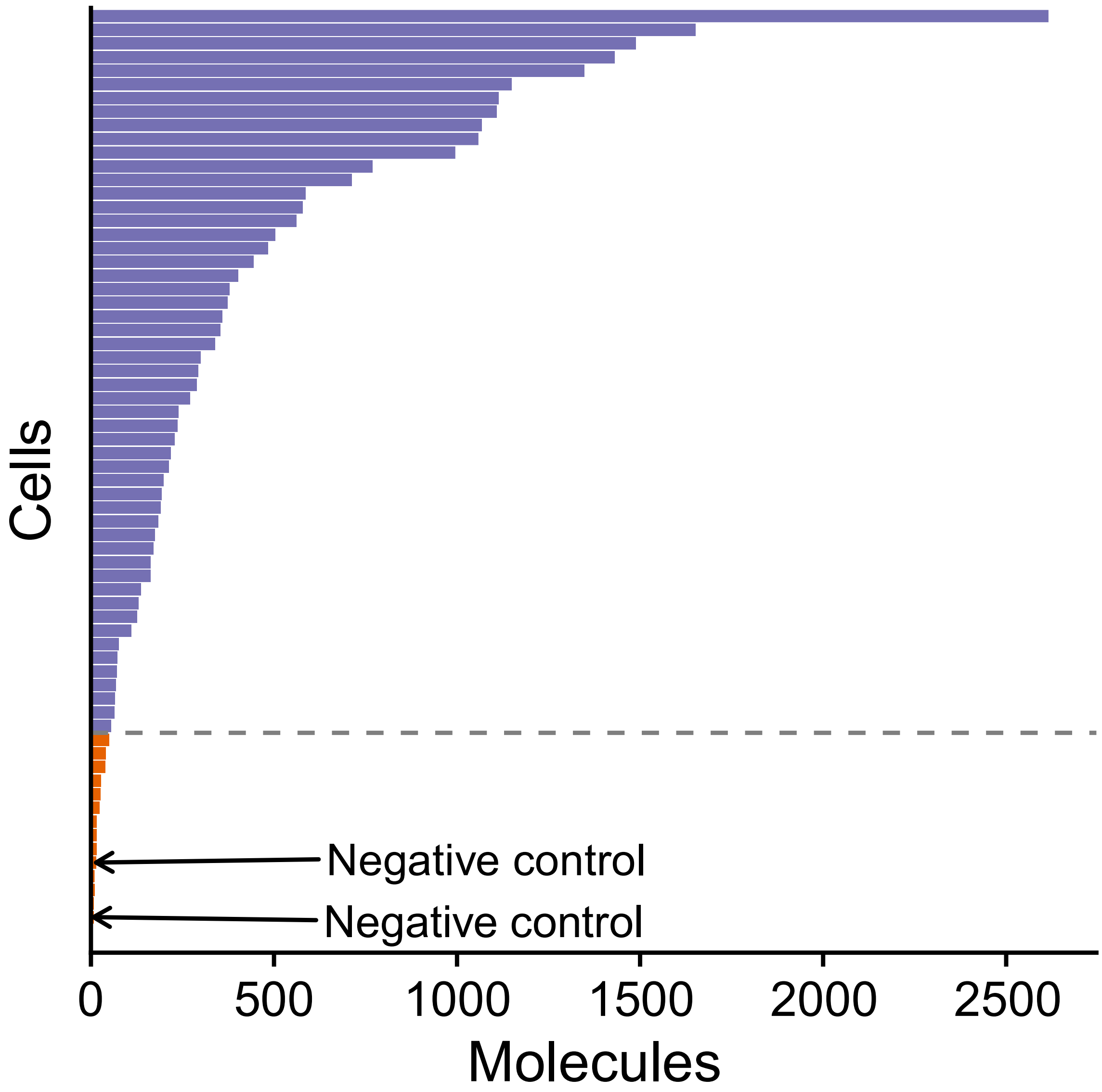

Supplementary Figure 11: Distribution of the number of miRNA molecules found per cell. The cells are ordered by decreasing number of miRNA molecules. All cells that did not meet the QC threshold of 50 molecules (grey dotted line) are shown in orange. The two empty negative controls are explicitly highlighted.

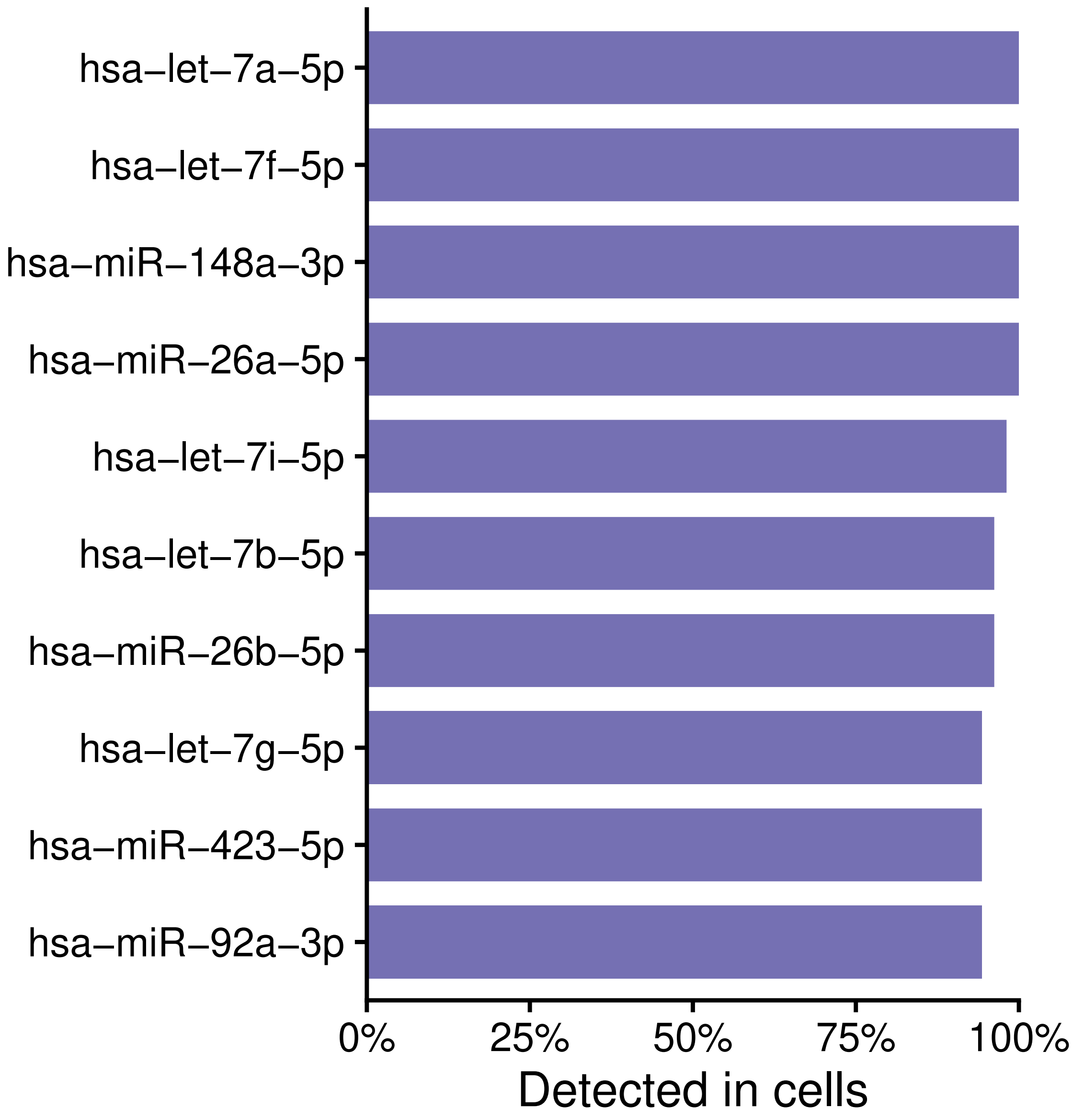

Supplementary Figure 12: Top 10 miRNAs that were detected in at least 90% of all cells. The miRNAs are ordered by decreasing proportion of cells they were detected in.

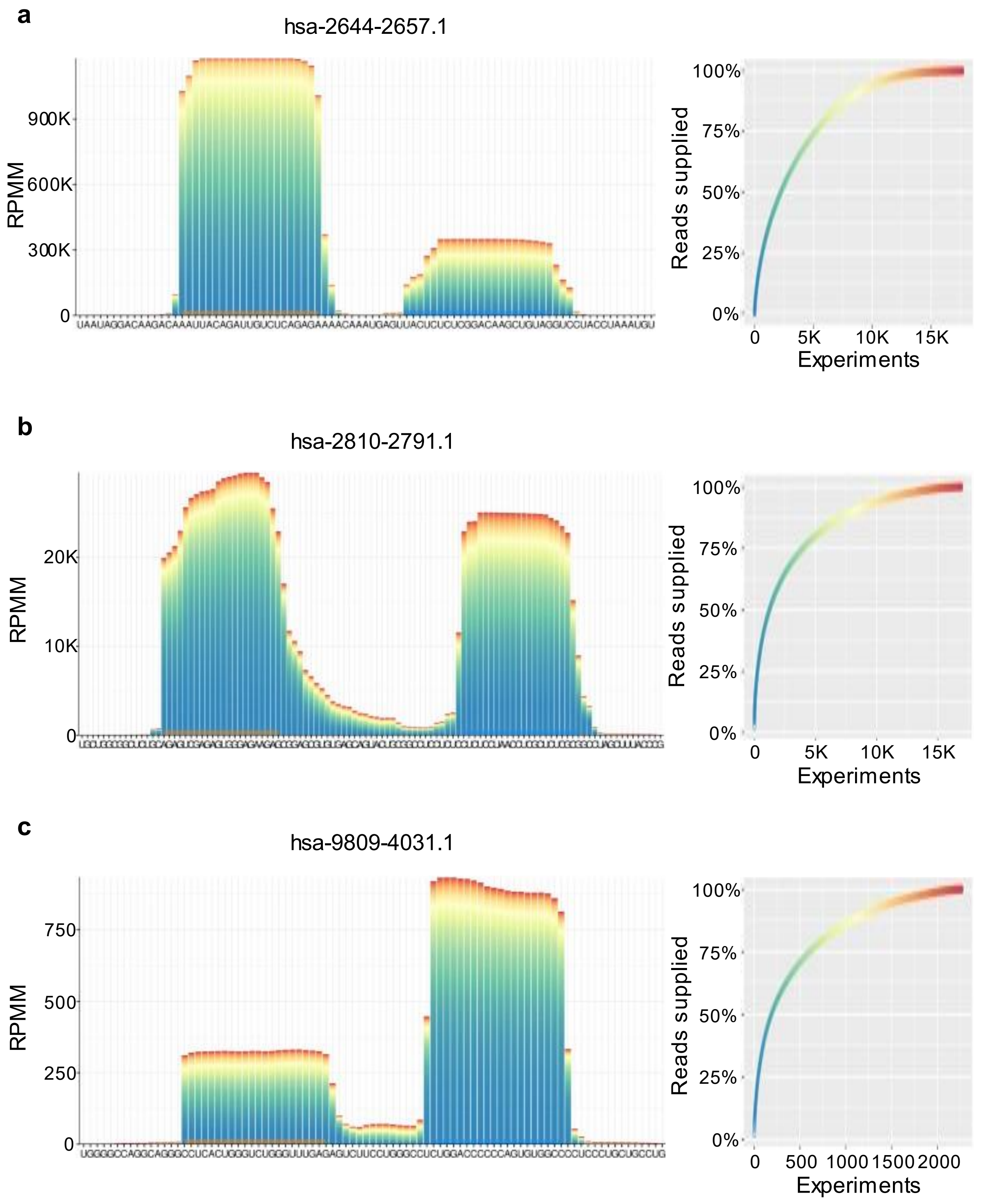

Supplementary Figure 13: Pileup plots obtained from miRCarta for three overlapping miRNA candidates. **a-c,** The bars are colored according to the experiments the reads were contributed from. The expression is shown as reads per million mapped (RPMM).

On the right, the distribution of the read proportion supplied by each experiment that detected the miRNA candidate is shown.

Supplementary Table 1: Comparison of the applied small RNA-Seq protocols. The table summarizes the different small RNA-Seq protocols tested with focus on differences in adapter modifications and concentrations, incubation times, and enzymes used.

protocol	protocol principle	basic protocol	3' adapter	5' adapter	concentration 3' adapter [µM]	ligation time 3' adapter	concentration 5' adapter [µM]	ligase addition 5' adapter	adapter dimer removal strategy	concentration RT primer [µM]	reverse transcriptase	PCR polymerase	number of PCR cycles
**SB**	sequential adapter ligation	SB	SB	SB	0.017	over night	0.09	T4 RNA ligase	exonuclease digest	0.2	SuperScript II	Phusion Hot Start II	13/13
**SB_4N**	sequential adapter ligation	SB	4N	4N	0.017	over night	0.09	T4 RNA ligase	exonuclease digest	0.2	SuperScript II	Phusion Hot Start II	13/13
**SB_C3**	sequential adapter ligation	SB	SB	C3	0.017	over night	0.09	T4 RNA ligase	exonuclease digest	0.2	SuperScript II	Phusion Hot Start II	13/13
**SB_CL**	sequential adapter ligation	SB	CL	CL	0.017	over night	0.09	T4 RNA ligase	exonuclease digest & chemically modified adapters	0.2	SuperScript II	Phusion Hot Start II	13/13
**SBN**	sequential adapter ligation	SBN	SB	SB	2	over night	1	T4 RNA ligase 1 ssRNA	exonuclease digest	5	SuperScript II	Phusion Hot Start II	13/13
**SBN_4N**	sequential adapter ligation	SBN	4N	4N	2	over night	1	T4 RNA ligase 1 ssRNA	exonuclease digest	5	SuperScript II	Phusion Hot Start II	13/13
**SBN_CL**	sequential adapter ligation	SBN	CL	CL	2	over night	1	T4 RNA ligase 1 ssRNA	exonuclease digest & chemically modified adapters	5	SuperScript II	Phusion Hot Start II	13/13
**CL**	sequential adapter ligation	CL	CL	CL	0.026	1.5 h	0.11	T4 RNA ligase	chemically modified adapters	0.018	SuperScript II	Phusion Hot Start II	13/13
**CL_16C**	sequential adapter ligation	CL	CL	CL	0.026	over night	0.11	T4 RNA ligase	chemically modified adapters	0.018	SuperScript II	Phusion Hot Start II	13/13
**CL_4N**	sequential adapter ligation	CL	4N	4N	0.026	1.5 h	0.11	T4 RNA ligase	none	0.018	SuperScript II	Phusion Hot Start II	13/13
**CL_Block**	sequential adapter ligation	CL	CL	Block	0.026	1.5 h	0.11	T4 RNA ligase	chemically modified adapters	0.018	SuperScript II	Phusion Hot Start II	13/13
**CL_C3**	sequential adapter ligation	CL	CL	C3	0.026	1.5 h	0.11	T4 RNA ligase	chemically modified adapters	0.018	SuperScript II	Phusion Hot Start II	13/13
**CL_Rand**	sequential adapter ligation	CL	Rand	CL	0.026	1.5 h	0.11	T4 RNA ligase	chemically modified adapters	0.018	SuperScript II	Phusion Hot Start II	13/13
**CL_SB**	sequential adapter ligation	CL	SB	SB	0.026	1.5 h	0.11	T4 RNA ligase	none	0.018	SuperScript II	Phusion Hot Start II	13/13
**CL_UMI6**	sequential adapter ligation	CL	CL	UMI6	0.026	1.5 h	0.11	T4 RNA ligase	chemically modified adapters	0.018	SuperScript II	Phusion Hot Start II	13/13
**CATS**	polyadenylation	CATS	n.a.	n.a.	n.a.	n.a.	n.a.	n.a.	none	unknown	Reverse Transcription Reagent of Diagenode Kit	PCR master mix of Diagenode Kit	24
**4N**	sequential adapter ligation	4N	4N	4N	0.111	2 h	0.08	T4 RNA ligase 1 ssRNA	exonuclease digest	0.053	SuperScript III	NEBNext Ultra II Q5	20
**4N_C3**	sequential adapter ligation	4N	4N	C3	0.111	2 h	0.08	T4 RNA ligase 1 ssRNA	exonuclease digest	0.053	SuperScript III	NEBNext Ultra II Q5	20
**4N_CL**	sequential adapter ligation	4N	CL	CL	0.111	2 h	0.08	T4 RNA ligase 1 ssRNA	exonuclease digest & chemically modified adapters	0.053	SuperScript III	NEBNext Ultra II Q5	20

Supplementary Table 2: Library concentrations of the spike-in experiments (stage 1).

protocol name	replicate	index sequence	library concentration [ng/µl]	sequencing run	comment
**SB_CL**	1	TGGTCA	0,618	1	
**SB_CL**	2	CACTGT	0,57	1	
**SB_CL**	3	ATTGGC	0,626	1	
**CL**	1	GATCTG	13,3	1	
**CL**	2	TCAAGT	2,98	1	
**CL**	3	CTGATC	6,34	1	
**CL**	4	CGTACG	4,75	4	
**CL**	5	CCACTC	2,12	4	
**CL**	6	GCTACC	2,26	4	
**CL_Block**	1	AAGCTA	22	1	
**CL_Block**	2	GTAGCC	21,2	1	
**CL_Block**	3	TACAAG	26,2	1	
**4N**	1	TTGACT	0,558	1	
**4N**	2	GGAACT	2,66	1	
**4N**	3	TGACAT	3,6	1	
**4N**	4	TTGACT	33,6	4	
**4N**	5	GGAACT	33,2	4	
**4N**	6	TGACAT	22,8	4	
**4N_CL**	1	GGACGG	0,342	1	
**4N_CL**	2	CTCTAC	0,41	1	library prep failed, sample not sequenced
**4N_CL**	3	GCGGAC	0,336	1	
**4N_CL**	4	TTTCAC	10,6	4	
**4N_CL**	5	GGCCAC	11,8	4	
**4N_CL**	6	CGAAAC	12,4	4	
**4N_C3**	1	TTTCAC	0,504	1	
**4N_C3**	2	GGCCAC	0,624	1	
**4N_C3**	3	CGAAAC	0,442	1	
**4N_C3**	4	CCGTCC	43,2	4	
**4N_C3**	5	GTAGAG	33	4	
**4N_C3**	6	GTCCGC	43,8	4	
**CL_16C**	1	CGTACG	4,54	1	
**CL_16C**	2	CCACTC	0,37	1	
**CL_16C**	3	GCTACC	0,36	1	
**CL_UMI6**	1	ATCAGT	0,356	1	
**CL_UMI6**	2	GCTCAT	0,428	1	
**CL_UMI6**	3	AGGAAT	0,392	1	
**CL_C3**	1	CTTTTG	0,392	1	
**CL_C3**	2	TAGTTG	0,42	1	
**CL_C3**	3	CCGGTG	0,358	1	
**CL_Rand**	1	CGTGAT	23,6	1	
**CL_Rand**	2	ACATCG	18,6	1	
**CL_Rand**	3	GCCTAA	13,4	1	
**CL_4N**	1	ATCGTG	26	1	
**CL_4N**	2	TGAGTG	23	1	
**CL_4N**	3	CGCCTG	21,4	1	
**CL_SB**	1	GCCATG	23,2	1	

**CL_SB**	2	AAAATG	24,8	1	
**CL_SB**	3	TGTTGG	7,96	1	
**SB_C3**	1	TCTGAG	2,62	1	
**SB_C3**	2	GTCGTC	2,52	1	
**SB_C3**	3	CGATTA	2,88	1	
**SB_4N**	1	ATTCCG	7,48	1	
**SB_4N**	2	AGCTAG	14,7	1	
**SB_4N**	3	GTATAG	12,7	1	
**SB_4N**	4	TGGTCA	23	4	
**SB_4N**	5	CACTGT	21,8	4	
**SB_4N**	6	ATTGGC	21,2	4	
**SBN**	1	CGTGAT	16,6	2	
**SBN**	2	ACATCG	16,1	2	
**SBN**	3	GCCTAA	12,2	2	
**SBN**	4	GATCTG	36,8	4	
**SBN**	5	TCAAGT	34	4	
**SBN**	6	CTGATC	30,4	4	
**SBN_CL**	1	TGGTCA	4,98	2	
**SBN_CL**	2	CACTGT	8,92	2	
**SBN_CL**	3	ATTGGC	9,54	2	
**SBN_CL**	4	AAGCTA	25,4	4	
**SBN_CL**	5	GTAGCC	31,4	4	
**SBN_CL**	6	TACAAG	34,6	4	
**SBN_4N**	1	GATCTG	43	2	
**SBN_4N**	2	TCAAGT	41,4	2	
**SBN_4N**	3	CTGATC	42,2	2	
**CATS**	1	AAGCTA	7,2	2	
**CATS**	2	GTAGCC	7,1	2	
**CATS**	3	TACAAG	6,58	2	
**SB**	1	CTTTTG	21,6	3	
**SB**	2	TAGTTG	22,4	3	
**SB**	3	CCGGTG	20,6	3	
**SB**	4	CGTGAT	19,6	4	
**SB**	5	ACATCG	22	4	
**SB**	6	GCCTAA	20	4	

Supplementary Table 3: Library concentrations of the MCF7 single cell equivalents experiments (stage 2).

protocol name	replicate	index sequence	library concentration [ng/µl]	sequencing run
**SB_4N**	1	TTGACT	2,44	2
**SB_4N**	2	GGAACT	2,7	2
**SB_4N**	3	TGACAT	2,6	2
**SB_4N**	4	CTTTTG	3,2	5
**SB_4N**	5	TAGTTG	3,34	5
**SB_4N**	6	CCGGTG	3,08	5
**CL**	1	GGACGG	1,43	2
**CL**	2	CTCTAC	1,62	2
**CL**	3	GCGGAC	1,42	2
**CL**	4	TCGGGA	1,27	5
**CL**	5	CTTCGA	1,25	5
**CL**	6	TGCCGA	1,25	5
**4N**	1	ATCAGT	0,88	2
**4N**	2	GCTCAT	39,6	2
**4N**	3	AGGAAT	7,7	2
**4N**	4	ATTCCG	21,4	5
**4N**	5	AGCTAG	21,4	5
**4N**	6	GTATAG	18,5	5
**4N_C3**	1	CTTTTG	48,4	2
**4N_C3**	2	TAGTTG	73,8	2
**4N_C3**	3	CCGGTG	53,6	2
**4N_C3**	4	TCTGAG	17,8	5
**4N_C3**	5	GTCGTC	8,06	5
**4N_C3**	6	CGATTA	17,5	5
**4N_CL**	1	ATCGTG	1,57	2
**4N_CL**	2	TGAGTG	1,05	2
**4N_CL**	3	CGCCTG	1,26	2
**4N_CL**	4	GCTGTA	8,84	5
**4N_CL**	5	ATTATA	9,54	5
**4N_CL**	6	GAATGA	10,4	5
**SBN**	1	CGTGAT	44,2	3
**SBN**	2	ACATCG	26,4	3
**SBN**	3	GCCTAA	36,2	3
**SBN**	4	ATCGTG	14,9	5
**SBN**	5	TGAGTG	13,9	5
**SBN**	6	CGCCTG	13	5
**SBN_CL**	1	TGGTCA	0,316	3
**SBN_CL**	2	CACTGT	0,41	3
**SBN_CL**	3	ATTGGC	0,34	3
**SBN_CL**	4	GCCATG	6,38	5
**SBN_CL**	5	AAAATG	5,7	5
**SBN_CL**	6	TGTTGG	7,84	5
**SB**	1	ATCGTG	0,428	3
**SB**	2	TGAGTG	0,434	3
**SB**	3	CGCCTG	0,442	3
**SB**	4	ATCAGT	1,94	5
**SB**	5	GCTCAT	2,1	5
**SB**	6	AGGAAT	2,04	5

Supplementary Table 4: Oligonucleotides. For all oligonucleotides, 100 µM stocks were prepared and either stored at -20°C (DNA oligos) or -80°C (RNA oligos).

oligo name	sequence	specification
**SB 3' adapter**	rApp-TGGAATTCTCGGGTGCCAAGG-ddC	rApp = preadenylated 5'end; ddC = dideoxycytidine
**CL 3' adapter**	rApp-T(MP)GGAATTCTCGGGTGCCAAGG-ddC	rApp = preadenylated 5'end; MP = methylphosphonate; ddC = dideoxycytidine
**Rand 3' adapter**	rApp-NNNNNNTCTCGGGTGCCAAGG-C3	rApp = preadenylated 5'end; N = A, T, G or C; C3 = spacer
**4N 3' adapter**	rApp-NNNNTGGAATTCTCGGGTGCCAAGG-ddC	rApp = preadenylated 5'end; N = A, T, G or C; ddC = dideoxycytidine
**SB 5' adapter**	NH2-rGrUrUrCrArGrArGrUrUrCrUrArCrArGrUrCrCrGrArCrGrArUrCrHrHrHrHrHrHrHrCrA	NH2 = aminolinker; r = ribonucleotide; H = A, U or C
**C3 5' adapter**	rGrUrUrCrArGrArGrUrUrCrUrArCrArGrUrCrCrGrArCrGrArUrCrHrHrHrHrHrHrHrArArUrUrCrCrA	r = ribonucleotide; H = A, U or C
**CL 5' adapter**	rGrUrUrCrArGrArGrUrUrCrUrArCrArGrUrCrCrGrArCrGrArUrCrHrHrHrHrHrHrHrCrA-2OMe	r = ribonucleotide; H = A, U or C; 2OMe = 2'-O-Methyl
**Block 5' adapter**	rGrUrUrCrArGrArGrUrUrCrUrArCrArGrUrCrCrGrArCrGrArUrCrHrHrHrHrHrHrCrArUrUrU	r = ribonucleotide; H = A, U or C
**UMI6 5' adapter**	rGrUrUrCrArGrArGrUrUrCrUrArCrArGrUrCrCrGrArCrGrArUrCrHrHrHrHrHrHrCrA-2OMe	r = ribonucleotide; H = A, U or C
**4N 5' adapter**	rGrUrUrCrArGrArGrUrUrCrUrArCrArGrUrCrCrGrArCrGrArUrCrNrNrNrN	r = ribonucleotide; N = A, U, G or C
**5.8S rRNA mask**	ATCGGCAAGCGACGCTCAGACAGGCGTAGCCCCGGGAGGAACCCGGGGCCGCAAGTGCGTTCGA AGTGTCGATGAT-TEG-biotin	
**RT primer**	Biotin-CCTTGGCACCCGAGAATTCCrA	r = ribonucleotide
**Rand RT primer**	Biotin-CCTTGGCACCCGAGA	
**Block RT primer**	Biotin-CCTTGGCACCCGAGAATTCCAAAATG	
**RP1 primer**	AATGATACGGCGACCACCGAGATCTACACGTTCAGAGTTCTACAGTCCGA	
**Index RPI1**	CAAGCAGAAGACGGCATACGAGATCGTGATGTGACTGGAGTTCCTTGGCACCCGAGAATTCCA	
**Index RPI2**	CAAGCAGAAGACGGCATACGAGATACATCGGTGACTGGAGTTCCTTGGCACCCGAGAATTCCA	
**Index RPI3**	CAAGCAGAAGACGGCATACGAGATGCCTAAGTGACTGGAGTTCCTTGGCACCCGAGAATTCCA	
**Index RPI4**	CAAGCAGAAGACGGCATACGAGATTGGTCAGTGACTGGAGTTCCTTGGCACCCGAGAATTCCA	
**Index RPI5**	CAAGCAGAAGACGGCATACGAGATCACTGTGTGACTGGAGTTCCTTGGCACCCGAGAATTCCA	
**Index RPI6**	CAAGCAGAAGACGGCATACGAGATATTGGCGTGACTGGAGTTCCTTGGCACCCGAGAATTCCA	
**Index RPI7**	CAAGCAGAAGACGGCATACGAGATGATCTGGTGACTGGAGTTCCTTGGCACCCGAGAATTCCA	
**Index RPI8**	CAAGCAGAAGACGGCATACGAGATTCAAGTGTGACTGGAGTTCCTTGGCACCCGAGAATTCCA	
**Index RPI9**	CAAGCAGAAGACGGCATACGAGATCTGATCGTGACTGGAGTTCCTTGGCACCCGAGAATTCCA	
**Index RPI10**	CAAGCAGAAGACGGCATACGAGATAAGCTAGTGACTGGAGTTCCTTGGCACCCGAGAATTCCA	
**Index RPI11**	CAAGCAGAAGACGGCATACGAGATGTAGCCGTGACTGGAGTTCCTTGGCACCCGAGAATTCCA	
**Index RPI12**	CAAGCAGAAGACGGCATACGAGATTACAAGGTGACTGGAGTTCCTTGGCACCCGAGAATTCCA	
**Index RPI13**	CAAGCAGAAGACGGCATACGAGATTTGACTGTGACTGGAGTTCCTTGGCACCCGAGAATTCCA	
**Index RPI14**	CAAGCAGAAGACGGCATACGAGATGGAACTGTGACTGGAGTTCCTTGGCACCCGAGAATTCCA	
**Index RPI15**	CAAGCAGAAGACGGCATACGAGATTGACATGTGACTGGAGTTCCTTGGCACCCGAGAATTCCA	
**Index RPI16**	CAAGCAGAAGACGGCATACGAGATGGACGGGTGACTGGAGTTCCTTGGCACCCGAGAATTCCA	
**Index RPI17**	CAAGCAGAAGACGGCATACGAGATCTCTACGTGACTGGAGTTCCTTGGCACCCGAGAATTCCA	
**Index RPI18**	CAAGCAGAAGACGGCATACGAGATGCGGACGTGACTGGAGTTCCTTGGCACCCGAGAATTCCA	
**Index RPI19**	CAAGCAGAAGACGGCATACGAGATTTTCACGTGACTGGAGTTCCTTGGCACCCGAGAATTCCA	
**Index RPI20**	CAAGCAGAAGACGGCATACGAGATGGCCACGTGACTGGAGTTCCTTGGCACCCGAGAATTCCA	
**Index RPI21**	CAAGCAGAAGACGGCATACGAGATCGAAACGTGACTGGAGTTCCTTGGCACCCGAGAATTCCA	
**Index RPI22**	CAAGCAGAAGACGGCATACGAGATCGTACGGTGACTGGAGTTCCTTGGCACCCGAGAATTCCA	
**Index RPI23**	CAAGCAGAAGACGGCATACGAGATCCACTCGTGACTGGAGTTCCTTGGCACCCGAGAATTCCA	
**Index RPI24**	CAAGCAGAAGACGGCATACGAGATGCTACCGTGACTGGAGTTCCTTGGCACCCGAGAATTCCA	
**Index RPI25**	CAAGCAGAAGACGGCATACGAGATATCAGTGTGACTGGAGTTCCTTGGCACCCGAGAATTCCA	
**Index RPI26**	CAAGCAGAAGACGGCATACGAGATGCTCATGTGACTGGAGTTCCTTGGCACCCGAGAATTCCA	
**Index RPI27**	CAAGCAGAAGACGGCATACGAGATAGGAATGTGACTGGAGTTCCTTGGCACCCGAGAATTCCA	

**Index RPI28**	CAAGCAGAAGACGGCATACGAGATCTTTTGGTGACTGGAGTTCCTTGGCACCCGAGAATTCCA	
**Index RPI29**	CAAGCAGAAGACGGCATACGAGATTAGTTGGTGACTGGAGTTCCTTGGCACCCGAGAATTCCA	
**Index RPI30**	CAAGCAGAAGACGGCATACGAGATCCGGTGGTGACTGGAGTTCCTTGGCACCCGAGAATTCCA	
**Index RPI31**	CAAGCAGAAGACGGCATACGAGATATCGTGGTGACTGGAGTTCCTTGGCACCCGAGAATTCCA	
**Index RPI32**	CAAGCAGAAGACGGCATACGAGATTGAGTGGTGACTGGAGTTCCTTGGCACCCGAGAATTCCA	
**Index RPI33**	CAAGCAGAAGACGGCATACGAGATCGCCTGGTGACTGGAGTTCCTTGGCACCCGAGAATTCCA	
**Index RPI34**	CAAGCAGAAGACGGCATACGAGATGCCATGGTGACTGGAGTTCCTTGGCACCCGAGAATTCCA	
**Index RPI35**	CAAGCAGAAGACGGCATACGAGATAAAATGGTGACTGGAGTTCCTTGGCACCCGAGAATTCCA	
**Index RPI36**	CAAGCAGAAGACGGCATACGAGATTGTTGGGTGACTGGAGTTCCTTGGCACCCGAGAATTCCA	
**Index RPI37**	CAAGCAGAAGACGGCATACGAGATATTCCGGTGACTGGAGTTCCTTGGCACCCGAGAATTCCA	
**Index RPI38**	CAAGCAGAAGACGGCATACGAGATAGCTAGGTGACTGGAGTTCCTTGGCACCCGAGAATTCCA	
**Index RPI39**	CAAGCAGAAGACGGCATACGAGATGTATAGGTGACTGGAGTTCCTTGGCACCCGAGAATTCCA	
**Index RPI40**	CAAGCAGAAGACGGCATACGAGATTCTGAGGTGACTGGAGTTCCTTGGCACCCGAGAATTCCA	
**Index RPI41**	CAAGCAGAAGACGGCATACGAGATGTCGTCGTGACTGGAGTTCCTTGGCACCCGAGAATTCCA	
**Index RPI42**	CAAGCAGAAGACGGCATACGAGATCGATTAGTGACTGGAGTTCCTTGGCACCCGAGAATTCCA	
**Index RPI43**	CAAGCAGAAGACGGCATACGAGATGCTGTAGTGACTGGAGTTCCTTGGCACCCGAGAATTCCA	
**Index RPI44**	CAAGCAGAAGACGGCATACGAGATATTATAGTGACTGGAGTTCCTTGGCACCCGAGAATTCCA	
**Index RPI45**	CAAGCAGAAGACGGCATACGAGATGAATGAGTGACTGGAGTTCCTTGGCACCCGAGAATTCCA	
**Index RPI46**	CAAGCAGAAGACGGCATACGAGATTCGGGAGTGACTGGAGTTCCTTGGCACCCGAGAATTCCA	
**Index RPI47**	CAAGCAGAAGACGGCATACGAGATCTTCGAGTGACTGGAGTTCCTTGGCACCCGAGAATTCCA	
**Index RPI48**	CAAGCAGAAGACGGCATACGAGATTGCCGAGTGACTGGAGTTCCTTGGCACCCGAGAATTCCA	

Supplementary Table 5: SCLC patient data. The table lists the age, sex, tumor staging, therapy information, smoking status, and number of CTCs detected with Cell Search of the seven SCLC patients included in this study.

patient	sex	age	staging	Ki67	therapy	number of CTCs detected with Cell Search	timepoint of blood draw	smoking status
**P 1**	female	60	TxN3M1c	N/A	1st line: Carboplatin/Etoposid (+Atezolizumab from cycle 3 on, followed by Atezolizumab maintenance after 6 cycles chemotherapy)	523	sample taken before start of 1st line therapy	smoker
**P 2**	male	60	T4N2M1c	80%	1st line: Carboplatin/Etoposid; 2nd line: Topotecan	921	sample taken before start of 2nd line therapy	ex-smoker
**P 3**	male	54	T2bN3M1c	90%	1st line: Carboplatin/Etoposid	14	sample taken before start of 1st line therapy	ex-smoker
**P 4**	male	72	T4N2M1b	N/A	1st line: Carboplatin/Etoposid	0	sample taken after 1st line therapy	smoker
**P 5**	female	69	T4N3M0	80%	1st line: Carboplatin/Etoposid (4 cycles); 2nd line Carboplatin/Etoposid (6 cycles); 3rd line: Topotecan (2 cycles)	273	sample taken before start of 1st line therapy	smoker
**P 6**	male	68	T4N1M1	70%	1st line: Carboplatin/Etoposid; 2nd line: Topotecan	129	sample taken before start of 1st line therapy	ex-smoker
**P 7**	female	66	T4N3M0	50-60%	1st line: Carboplatin/Etoposid; 2nd line: Topotecan	0	sample taken after 1st line therapy	ex-smoker
