# Supplementary material for: Single-cell microRNA sequencing method comparison and application to cell lines and circulating lung tumor cells
Source: Nat Commun. 2021 Jul 14;12:4316. doi: 10.1038/s41467-021-24611-w (PMC8280203; doi:10.1038/s41467-021-24611-w)
Supplement: Supplementary file 2 — Description of Additional Supplementary Files [file 41467_2021_24611_MOESM2_ESM.docx]

**Description of Additional Supplementary Files**

File Name: Supplementary Data 1

Description: Sequences and corresponding miRNAs present in the miRXplore spike-in.

File Name: Supplementary Data 2

Description: Mapping statistics of all tested spike-in protocols. The table shows the number of sequenced reads per library, before and after QC, the proportion of reads mapped to miRXplore, uniquely mapped, multimapped and unmapped reads to hg38. Additionally, the proportion of reads mapping to annotated genomic features (e.g., miRNA, protein-coding genes, intergenic regions, or lncRNA) is listed.

File Name: Supplementary Data 3

Description: Detection rate, sequence composition and minimum free energy (MFE) of secondary structure of all miRXplore spike-in sequences.

File Name: Supplementary Data 4

Description: Mapping statistics for the MCF7 single cell equivalent experiments. The table shows the number of sequenced reads per library, before and after QC, the proportion of uniquely mapped, multimapped and unmapped reads to hg38. Additionally, the proportion of reads mapping to annotated genomic features (e.g., miRNA, protein-coding genes, intergenic regions, or lncRNA) is listed.

File Name: Supplementary Data 5

Description: Mapping statistics for the single cell samples of 8 cell lines. The table shows the number of sequenced reads per library, before and after QC, the proportion of uniquely mapped, multimapped and unmapped reads to hg38. Additionally, the proportion of reads mapping to annotated genomic features (e.g., miRNA, protein-coding genes, intergenic regions, or lncRNA) is listed.

File Name: Supplementary Data 6

Description: Mapping statistics for the SCLC patient CTCs. The table shows the number of sequenced reads per library, before and after QC, the proportion of uniquely mapped, multimapped and unmapped reads to hg38. Additionally, the proportion of reads mapping to annotated genomic features (e.g., miRNA, protein-coding genes, intergenic regions, or lncRNA) is listed.

File Name: Supplementary Data 7

Description: List of all quantified miRNAs expressed in CTCs of SCLC patients showing the mean expression, standard deviation, median expression and number of cells a miRNA was expressed in.

File Name: Supplementary Data 8

Description: Enrichment results obtained with miEAA 2.0 in SCLC patients. Exact p-values were computed by the gene set enrichment analysis implementation of miEAA for each enrichment and FDR adjusted, separately for each database.
